# Into the Woods or a Stroll in the Park: How Virtual Contact with Nature Impacts Positive and Negative Affect

**DOI:** 10.3390/ijerph14070786

**Published:** 2017-07-14

**Authors:** Elizabeth McAllister, Navjot Bhullar, Nicola S. Schutte

**Affiliations:** School of Behavioural, Cognitive and Social Sciences, University of New England, Armidale 2351, Australia; libbymcallister@hotmail.com (E.M.); nschutte@une.edu.au (N.S.S.)

**Keywords:** wild nature, urban nature, restorativeness, affect, mental health

## Abstract

This study examined the effects of virtual contact with nature on positive and negative affect, and investigated the psychological process of perceived restorativeness as a mediator of this relationship. A sample of 220 Australians aged between 18 and 75 years (*M* = 49.07, *SD* = 14.34, female = 72%) participated in the study. Participants were randomly allocated to one of the three experimental conditions experienced through video presentations: (1) ‘wild’ nature, (2) ‘urban’ nature, and (3) non-nature control. They then completed measures of perceived restorativeness as well as positive and negative affect. Compared to the non-nature control condition, the experience of wild nature resulted in significantly higher levels of positive affect and lower levels of negative affect. The experience of urban nature resulted in significantly lower levels of negative affect only compared to the non-nature control video. Experience of wild and urban nature resulted in greater perceptions of restorativeness as compared to the non-nature control video. Restorativeness was a significant underlying psychological mediating path through which nature experience exerted its influence on affect. These results have the potential to inform nature-based green care interventions for mental health as well as for urban planning to maximize beneficial effects of natural environments.

## 1. Introduction

Contact with nature has been linked to enhanced mood [1,2]. This is consistent with the Biophilia hypothesis [3] which suggests that humans have an innate desire to affiliate with other living things and that they derive well-being from experiencing this affiliation. The mechanism by which contact with different natural systems (e.g., wild and urban nature) improves affect still remains unclear. Understanding the process by which nature exposure exerts its influence is important; first to inform clinical practice, as increased contact with nature potentially provides an accessible, cost-effective intervention for improving mood [4], and second to support urban planning that maximizes the effectiveness of natural areas in supporting human well-being [5].

### 1.1. Contact with Nature

Nature is commonly thought of as wild plants, animals, ecosystems, and landscapes in contrast to human built environments and natural places impacted by human activity. However, in the modern world, nature exists on a spectrum from places with little evidence of human activity to small parks in highly urbanized areas [6]. In ecology and environmental psychology, natural features that are partly the products of human activity, such as urban parks and back yards, are widely considered to also represent nature [7,8,9]. 

In the present study, contact with nature was conceptualized as a person experiencing nature, including nature influenced by humans, by viewing and hearing the sounds of nature in video or still images [10,11].

### 1.2. Relationship between Contact with Nature and Affect

A meta-analysis of 32 studies examined the effect of contact with nature on positive affect and negative affect, and found a moderate increase in positive affect and a smaller but still significant decrease in negative affect for participants who had contact with nature compared to those who did not [2]. Another meta-analysis of 17 studies comparing self-reported emotions after undertaking physical activity in a natural versus a non-natural environment found clear beneficial impacts of nature compared to non-nature on levels of anger, sadness, and fatigue [1]. This meta-analysis found an improvement from before to after contact with nature in levels of energy, anxiety, anger, sadness, and fatigue. A large cross-sectional study in the United Kingdom using a smartphone app titled “Mappiness” also found that participants responding to questions in outdoor settings and natural habitats were significantly happier than participants in urban environments [12]. In a cross-sectional study [13], a negative association was found between green space around residents’ homes and stress levels (as measured by saliva cortisol levels and self-report). 

Many experimental studies have exposed participants to virtual nature in the form of photographs and videos, due to the difficulties of taking participants outdoors and the fact that real natural settings feature potential confounding variables such as differing light, temperature, smells, and sounds. While the effects of nature are more pronounced when real-life nature is encountered, previous research has shown that virtual nature has similar, if weaker, effects on a range of psychological variables [10,11,14,15,16,17]. 

Studies making a direct comparison between the effects of “wild nature” (surrounds where human influence is not evident) versus “urban nature” (surrounds with clear human influence) have found varying effects [5,18]. Greater actual and perceived biodiversity have been found to increase the effect of nature walks on psychological well-being [19,20,21]. Another study [8] found that perceived naturalness and perceived restorativeness of the surrounds interact to enhance positive affect after an outdoor group walk. Such results indicate a positive effect for areas that are perceived as wild nature compared to urban nature. On the other hand, a meta-analytic study [2] found no significant differences in positive and negative affect between contact with wild nature and manicured nature. Similarly, a study examining the well-being benefits of contact with wild woodland, tended woodland, and parkland found no significant differences among the three [22]. 

There is evidence that contact with any nature may be more useful in increasing positive emotion than in reducing negative emotion [2]. Evidence from three experimental studies suggested a significant connection between contact with nature and increased levels of positive emotion, while differences in negative emotion were found to be non-significant [11]. A similar finding was observed in a sample of individuals diagnosed with Major Depressive Disorder [23]. However, it is still unclear how virtual experience of a wild or urban nature will differentially impact positive and negative affect, respectively compared with a non-nature control. 

### 1.3. Relationship between Contact with Nature and Restorativeness

The mechanism by which nature influences affective states is an active area of research. While often investigated, it appears that physical exercise and social contact cannot fully explain the beneficial effects of nature [1]. Attention restoration theory [7,24] has attracted much research effort as an explanation for nature’s beneficial effect on positive and negative affect. This theory proposes that directed attention to a task can only be sustained for so long before fatigue is experienced and attention lapses. It further posits that restoration of attentional capacity can be achieved in environments that absorb one’s attention seemingly effortlessly, a process termed fascination [24]. 

A number of experimental studies have compared participants’ attentional capacity after viewing photographs of, or visiting, natural versus urban places. These studies provide evidence of the attentional restoration benefits of even a brief interaction with nature, including virtual nature [25,26,27,28]. Additionally, many studies have documented the perceived restorativeness of a natural place using self-reporting methods [19,29,30]. A study of a sample of 319 young adult students in New Zealand found that the level of fascination in nature mediated the relationship between time in nature and positive affect [31]. Experimental research suggests that virtual natural environments impact restorativeness [16], and a greater perception of restorativeness is found to be associated with more positive affect and less negative affect [15,26]. Therefore, restorativeness might be the path that explains the effect of nature experience on affect. 

### 1.4. The Current Study

Using a psychological lens, the current study built on and extended previous research [2,8,11] in two important ways. First, this study experimentally compared the effects of virtual contact with wild nature and urban nature on positive and negative affect, respectively. To our knowledge, this direct comparison of virtual ‘wild’ nature and ‘urban’ nature experience and their differential impacts on positive and negative affect, respectively, have not been experimentally investigated. Based on previous research [16], we predicted that exposure to virtual wild nature compared with exposure to virtual urban nature would result in more positive affect and less negative affect, and greater perceived restorativeness. In addition, both virtual wild and urban nature experience would result in more positive affect and less negative affect, and a greater perception of restorativeness compared to exposure to a non-nature control.

Furthermore, the present study extended previous research by examining the role of perceived restorativeness as a mediating underlying psychological process explaining the relationship between the virtual nature experience and positive and negative affect. We predicted that the nature experience would influence perceived restorativeness, which in turn would influence positive and negative affect, respectively. More specifically, we predicted that the nature experience would result in a greater perception of restorativeness, which in turn would result in more positive affect and less negative affect. 

## 2. Method

### 2.1. Participants and Procedure

Two hundred and twenty Australian residents ranging in age from 18 to 75 years (Mean age = 49.07, *SD* = 14.34, female = 72%) completed the present study. The majority of the sample (76%) had completed a Bachelor or higher degree. 

The study was carried out using the Qualtrics research platform (Qualtrics, Provo, UT, USA). Before data collection, approval by the institutional human research ethics review committee (HREC, approval number HE15-129) was obtained. Participants were recruited from the general population. Invitations to participate were sent by email to social networks of the researchers. The invitation to participate was also included in the email newsletter of the Australian Psychological Society, and circulated to members of two outdoor activity clubs in Queensland, Australia. All participants were volunteers. Participants were invited to access the study anonymously via Qualtrics. Participants were randomly assigned to one of the three video experimental conditions and then completed measures of perceived restorativeness, positive and negative affect, as well as manipulation check, covariate (depressive symptoms), and demographic variables. Respondents did not receive any reimbursement or incentive for their participation.

### 2.2. Experimental Manipulation and Design 

Three experimental videos were created for the study to operationalize contact with nature (or a non-nature control) corresponding to the experimental conditions in this study. Participants were randomly allocated to wild nature (*n* = 72), urban nature (*n* = 76), and non-nature control (*n* = 72) conditions.

The videos were filmed in South East Queensland, Australia to create naturalistic representations of the nature and non-nature settings. Each video was shot in sunny Autumn weather during the middle of the day, and after the editing process displayed between 2 min 20 s and 2 min 35 s of footage with sound. Video and still images of approximately this duration have been found to be effective in eliciting affective responses to nature and other stimuli [32,33,34], and were found to be of optimal length whereby participants are more likely to watch the video in full, while still exerting a significant effect (see Section 3). 

Each video contained four or five scenes of roughly equal length, and panned slowly and horizontally within each scene. No coastal scenes were included, and inland bodies of water did not feature prominently. Footage of people was not included except where they were in the distance and unidentifiable. No explanatory text or music was included and no legible text was visible. The URLs for the videos are provided in Appendix A. 

The wild nature video featured rainforest and dry sclerophyll forest scenes filmed in Mount Glorious, Brisbane, Australia. A wide range of native tree, epiphyte, and understorey species was included. Birdsong was clearly audible throughout. Gravel walking tracks were the only evidence of human activity. The urban nature video featured public parks and gardens in urban contexts in the Brisbane suburbs of Sandgate and Boondall. Scenes included nature in the form of garden beds, birds, trees, and shrubs, including non-native species, while indicating the urban context through the presence of buildings, roads, cars and buses, lamp posts, concrete pathways, and vehicle sounds. People were sometimes visible in the distance. Finally, the non-nature control condition video showed urban built environments in the suburbs of South Brisbane and Taigum, and included minimal plants or animals. Concrete surfaces, roadways, buildings, and cars were visually dominant. Sounds were primarily road and aircraft noise. People were sometimes visible in the distance.

### 2.3. Measures

In addition to the demographic information (age, sex, education), we assessed the constructs of interest through the following measures. All Cronbach’s alphas (αs) reported, used to assess internal consistency of scales, are for the present study. 

#### 2.3.1. Perceived Restorativeness

The 26-item Perceived Restorativeness Scale (PRS) [15,35] is a self-report measure of how restorative an environment is perceived to be, in the context of attention restoration theory [7]. The PRS is scored on a seven-point scale ranging from 0 (not at all) to 6 (completely). Example items include “Being here is an escape experience” and “This place has fascinating qualities”. Participants were instructed to answer while thinking about the place in the video they had just watched, and items were reworded accordingly (e.g., “Being there is an escape experience”). (α = 0.97).

#### 2.3.2. Affect

The Positive and Negative Affect Schedule (PANAS) [36] was used to assess both positive and negative mood. The PANAS comprises two 10-item scales for positive affect and negative affect, respectively. Respondents are shown emotion-related words (e.g., “excited” for positive affect, and “upset” for negative affect) and asked to indicate to what extent they feel that way “right now—that is, at the present moment”. Responses are measured on a five-point scale ranging from 1 (very slightly or not at all) to 5 (extremely). (α_positive affect_ = 0.91, α_negative affect_ = 0.82).

#### 2.3.3. Manipulation Check and Covariate

A manipulation check item asked participants to nominate on a four-point scale whether they had watched all, half, a quarter, or none of the video presented. Previous research found that depressed mood was strongly associated with positive affect and negative affect [37], therefore, we assessed participants’ levels of depressive symptoms to control for this confounding variable. We used a 7-item depression subscale of the Depression Anxiety Stress Scales (DASS-21) [38]. The measure is assessed on a four-point scale from 0 (did not apply to me at all) to 3 (applied to me very much or most of the time). Scores on the scale are summed and then doubled for comparison with DASS-42 [38]. (α = 0.89). 

### 2.4. Statistical Analyses

Pearson’s correlation coefficients were used to examine bivariate relationships between key study variables. Analyses of covariance were conducted to examine group differences on positive and negative affect, and perceived restorativeness. Mediation analyses were conducted using the regression-based PROCESS macro (Model 4) [39] to examine the mediating effect of perceived restorativeness between the experience of nature and positive and negative affect, respectively. 

## 3. Results

### 3.1. Data Screening

A total of 274 participants commenced the survey and 54 cases (20%) were excluded; 48 participants did not proceed beyond the experimental manipulation (video) and six participants reported not watching the video and, therefore, failed to meet the manipulation check criterion. The remaining sample of 220 was used in the present study. 

### 3.2. Preliminary Analyses

Means, standard deviations, and intercorrelations of the study variables are presented in Table 1. Variables were normally distributed with the exception of negative affect; negative affect had a positive skew and a total sample mean negative affect score of 12.04, well below the mean of 16.00 found in a normative study of PANAS scores in a non-clinical population [40]. As expected, restorativeness was significantly associated with greater positive affect and lower levels of negative affect. There were also significant associations in the expected direction between positive and negative affect, and depressive symptoms, with the exception that higher positive affect was not significantly associated with lower negative affect. 

There were no significant differences between men and women on positive affect, *t*(218) = 1.50, *p* = 0.135, negative affect, *t*(218) = 0.74, *p* = 0.462, and perceived restorativeness, *t*(218) = 0.92, *p* = 0.361. Bivariate correlations were significant between age and positive affect, *r*(218) = 0.21, *p* < 0.01 and negative affect, *r*(218) = −0.30, *p* < 0.01, with older participants reporting more positive affect and less negative affect. Age was not significantly related to perceived restorativeness, *r*(218) = 0.04, *p* = 0.538, and there were also no significant experimental group differences in age, *F*(2, 217) = 0.44, *p* = 0.644.

### 3.3. Analyses of Covariance (ANCOVAs)

Three one-way ANCOVAs were used to examine the effect of experimental manipulation on perceived restorativeness and positive and negative affect. The experimental condition was used as the independent variable (IV), while positive affect, negative affect, and restorativeness were used as dependent variables (DVs), with depressive symptoms serving as a covariate. Univariate analyses revealed that there were significant group differences on positive affect, *F*(2, 216) = 5.29, *p* = 0.006, partial η^2^ = 0.05, a small effect size; negative affect, *F*(2, 215) = 5.51, *p* = 0.005, partial η^2^ = 0.05, a small effect size; and perceived restorativeness, *F*(2, 216) = 60.53, *p* < 0.001, partial η^2^ = 0.36, a large effect size [41]. 

Table 2 summarizes the covariate-adjusted means and standard errors for each DV by condition. For positive affect, the mean score for the wild nature condition was significantly higher than the mean score for the urban nature condition and the non-nature control condition. There was no significant difference between urban nature and non-nature control conditions on positive affect. Therefore, the effect of urban nature on positive affect was not tested in the subsequent mediation analysis for the relationship between urban nature experience and positive affect.

The wild and urban nature conditions resulted in significantly lower mean negative affect as compared to the non-nature control, however, the two nature conditions were not significantly different from each other. As expected, the wild nature experience resulted in significantly greater perceived restorativeness than the urban nature and non-nature control conditions, and the urban nature experience led to a greater perception of restorativeness than that of the non-nature condition. 

### 3.4. Mediation Analyses

To test the hypothesis that perceived restorativeness would mediate the effect of nature experience on positive and negative affect, respectively, we conducted three sets of regression-based mediation analyses using the PROCESS model [39]. This approach uses accelerated bootstrapped bias-corrected 95% confidence intervals (BCa CIs) for assessing the significance of indirect paths. For all analyses, the IV was created using a dummy coded variable: participants in the relevant nature (wild or urban) condition were assigned a value of 1 and participants in the control (non-nature) condition were assigned a value of 0; perceived restorativeness served as a two mediator, and depressive symptoms served as a covariate. 

**Figure 1:**
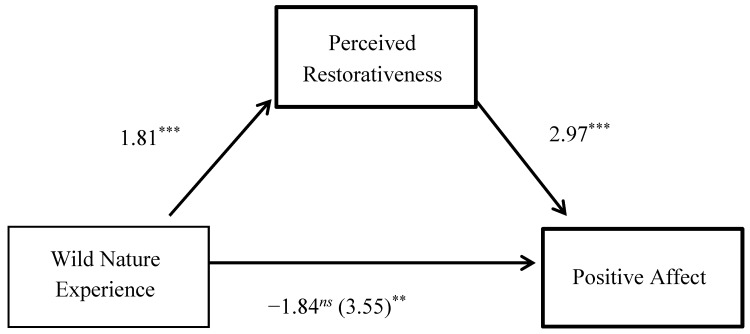
Perceived restorativeness as a mediator of the relationship between the virtual wild nature experience and positive affect. All paths are unstandardized beta coefficients [39]. The value in parenthesis represents the relationship between the wild nature experience and positive affect prior to controlling for perceived restorativeness. *N* = 144, ** *p* < 0.01, *** *p* < 0.001, *ns* = not significant.

For the first analysis, summarized in Figure 1, contact with wild nature was the IV and positive affect was the DV. Overall, the model explained 26% of the variance in positive affect, *F*(3, 140) = 16.41, *p* < 0.001. The effect of the wild nature experience on positive affect was mediated by perceived restorativeness, after controlling for depressive symptoms. The 95% BCa CIs for the indirect effect of 5.39 (*SE* = 1.10) ranged from 3.42 to 7.75 for perceived restorativeness, and a Sobel test of *z* = 4.61, *p* < 0.001 indicated a significant mediation. 

For the second analysis, contact with wild nature served as the IV and negative affect as the DV. Overall, this model explained 12% of the variance in negative affect, *F*(3, 140) = 6.40, *p* < 0.001. The results revealed that the effect of the wild nature experience on negative affect was unrelated to perceived restorativeness, after controlling for depressive symptoms. The 95% BCa CIs for the indirect effect of −0.48 (*SE* = 0.40) ranged from −1.32 to 0.27, and a Sobel test of *z* = −1.19, *p* = 0.24 suggested a statistically non-significant mediation.

**Figure 2:**
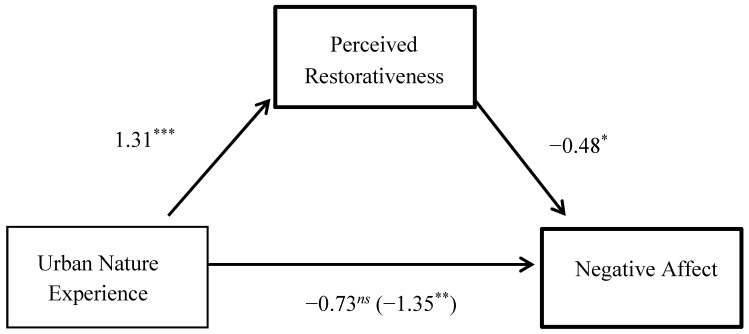
Perceived restorativeness as a mediator of the relationship between the virtual urban nature experience and negative affect. All paths are unstandardized beta coefficients [39]. The value in parenthesis represents the relationship between the wild nature experience and positive affect prior to controlling for perceived restorativeness. *N* = 148, * *p* < 0.05, ** *p* < 0.01, *** *p* < 0.001, *ns* = not significant.

The final analysis, summarized in Figure 2, used contact with urban nature as the IV and negative affect as the DV. Overall, this model explained 18% of the variance in negative affect, *F*(3, 144) = 6.82, *p* < 0.001. The effect of the urban nature experience on negative affect was mediated by perceived restorativeness, after controlling for depressive symptoms. The 95% BCa CI for the indirect effect of −0.62 (*SE* = 0.31) ranged from −1.32 to −0.08, and a Sobel test of *z* = −2.00, *p* = 0.045.

## 4. Discussion

The present study compared the impact of virtual contact with wild nature and urban nature to a non-nature control on positive and negative affect as well as perceived restorativeness. It also examined whether perceptions of restorativeness might be the path that links the relationship between virtual contact with nature and affect.

### 4.1. Effects of Contact with Wild and Urban Nature

The results add new detail to the understanding of well-being outcomes after virtual contact with different types of nature. Contact with wild nature resulted in significantly more positive affect benefit compared to contact with urban nature and the non-nature control condition. Contrary to previous research [2,42], virtual contact with urban nature was not significantly different from the non-nature control in eliciting positive affect. The present results suggest that only the wild nature experience enables elicitation of positive emotional states such as excitement, strength, and inspiration (as assessed by the positive affect subscale of PANAS) [36]. 

The results for negative affect suggest that virtual contact with either wild nature or urban nature significantly decreased negative affect, compared to a non-nature control, which is consistent with previous research [22]. There was no significant difference between the virtual experience of a wild or urban nature on negative affect. These results suggest that urban nature is as effective as wild nature in the alleviation of negative affect. This is a useful finding in the development of evidence for nature therapy. If contact with an urban park is as efficacious in easing feelings of distress, shame, or anxiety as travelling to a national park, more people may be able to experience the benefit of nature in reducing negative affect more often. 

The present study found small effect sizes for group differences in positive affect and negative affect, which is consistent with previous research [2,11]. The results of the present study indicated that exposure to virtual nature for 2–3 min can produce significant improvements in affective states. Our results add further evidence to the known beneficial effects of nature experience in improving mood. More specifically, it provides evidence of only wild nature experience increasing positive affect and either wild or urban nature experience lessening negative affect compared to a non-nature control. This finding has practical implications, as viewing short virtual nature videos could provide easily accessible and low-cost intervention to improve mood and mental health.

### 4.2. Mediation Analyses

For the relationship between virtual contact with wild nature and positive affect, the results support the hypothesis that perceived restorativeness is a path, or mediator, explaining this relationship. This is consistent with previous research showing perceived restorativeness as a path that provides a link between nature experience and affect [16,31], and provides further evidence that restoration of attention can increase positive affect. Perceived restorativeness also mediated the relationship between contact with urban nature and negative affect. This suggests that the mechanisms for the well-being effect of nature varies depending on the type of nature and is not the same for positive affect and negative affect.

### 4.3. Limitations and Future Research

The present study had some limitations that need to be considered when interpreting the current findings. Participants in the study sample reported low levels of negative affect. This floor effect may have not allowed a full test of the impact of nature in alleviating negative affect. Future work in this area may benefit from a process to induce negative affect prior to contact with nature, as has been used in some previous studies [43]. In investigating the impact of nature on negative affect, future research might also focus on populations of individuals high in negative affect, such as those experiencing anxiety. In the present study, we did not measure positive and negative affect at baseline, therefore, it was not possible to investigate the change in positive and negative affect in response to a virtual experience of wild or urban nature. Nevertheless, the random assignment of participants to different experimental conditions ensures no such systematic influences on our results. 

The specific operationalization of constructs used in the present study may also represent some limitations that can be addressed by future research. Measuring attentional capacity directly with cognitive tests, rather than through a self-report measure of restorativeness, would potentially improve validity. The videos used for the experimental manipulation were constructed to represent wild nature, urban nature, and a non-nature urban control condition. Each represented condition is in an Australian setting, therefore, replication of results with other nature and urban settings would increase confidence in the generalizability of the results. 

Studies which integrate consideration of eudaimonic and hedonic well-being may be useful in generating a more complete picture of psychological benefits of nature contact. The benefit of nature for eudaimonic well-being is an active area of research [44], but has attracted less research effort to date than subjective well-being. Future studies may also investigate the well-being effects of environments such as beaches, coral reefs, riverine areas, and alpine areas, as research into the psychology of contact with ecosystems other than forests and woodlands is rare [45]. Investigating benefits of nature contact for those diagnosed with mood or other psychological disorders is also a valuable research direction supporting clinical applications.

## 5. Conclusions

The findings of the present study indicate that even brief, virtual nature experience can enhance affect, and adds to the evidence of the benefits of increased green space in urban areas to reduce negative emotional states, thus promoting mental health. These findings provide support for attention restoration theory, with evidence that improvements in mood resulting from nature experience may be facilitated by the restorative impact of nature on attentional capacity.

## Figures and Tables

**Table 1:** Bivariate correlations, means, and standard deviations (SD) of key study variables.

Variables	1	2	3	4
**1. Perceived Restorativeness**	-	0.41 ***	−0.17 *	0.09
**2. Positive affect**		-	−0.09	−0.19 **
**3. Negative affect**			-	0.34 ***
**4. Depressive symptoms**				-
***M***	3.42	27.43	12.04	5.66
***SD***	1.26	8.26	2.85	6.04

* *p* < 0.05, ** *p* < 0.01, *** *p* < 0.001 (two-tailed).

**Table 2:** Covariate-adjusted means (*M*) and standard errors (*SE*) for key study variables for experimental conditions.

Condition	Positive Affect		Negative Affect		Perceived Restorativeness	
	*M*	*SE*	*M*	*SE*	*M*	*SE*
Wild nature (*n* = 72)	29.92 ^a^	0.94	11.77 ^a^	0.31	4.18 ^a^	0.12
Urban nature (*n* = 76)	25.88 ^b^	0.92	11.51 ^a^	0.30	3.69 ^b^	0.11
Non-nature control (*n* = 72)	26.58 ^b^	0.94	12.87 ^b^	0.31	2.38 ^c^	0.12

Means in each column with different superscripts (a, b, c) are significantly different from each other, using Sidak correction (*p* < 0.05).

## Appendix A

Wild nature video URL: https://www.youtube.com/watch?v=qHZ3rV6TzMs

Urban nature video URL: https://www.youtube.com/watch?v=RZMazVelWH4

Non-nature control video URL: https://www.youtube.com/watch?v=4nfG3xo3mBU
